# Semiautomatic assessment of endothelial density and morphology in organ-cultured corneas — potential predictors for transplantation suitability and clinical outcome?

**DOI:** 10.1007/s00417-023-06079-0

**Published:** 2023-04-28

**Authors:** Filip Filev, Mathias Stein, Maximilian Schultheiss, Antonia D. E. Fitzek, Jana Feuerstake, Oliver Engel, Olaf J. C. Hellwinkel

**Affiliations:** 1 Department of Ophthalmology, University Medical Center Hamburg-Eppendorf, Martitistr. 52, 20246 Hamburg, Germany; 2 Department of Ophthalmology, Werner Forssmann Hospital, Eberswalde, Germany; 3 Department of Forensic Medicine, University Medical Center Hamburg-Eppendorf, Martitistr. 52, 20246 Hamburg, Germany; 4 Department of Urology, Asklepios Klinikum Harburg, Eißendorfer Pferdeweg 52, 21075 Hamburg, Germany

**Keywords:** Corneal endothelium, Morphology, Cell density, Clinical outcome

## Abstract

**Background:**

The quality of the endothelial cell layer is a major criterion for the approval of organ-cultured human donor-corneas for transplantation. We wanted to compare the predictive capacities of initial endothelial density and endothelium cell morphology for the approval of donor corneas for transplantation and for the clinical outcome after transplantation.

**Methods:**

The endothelial density and endothelium morphology in organ culture were examined by semiautomatic assessment of 1031 donor corneas. We performed a statistical analysis for correlations of donor-data and cultivation parameters regarding their predictive capacities for the final approval of donor corneas for transplantation and the clinical outcome of 202 transplanted patients.

**Results:**

Corneal endothelium cell density proved to be the only parameter with a certain predictive capacity with regard to the final decision, whether donor corneas are suitable for transplantation — however, the correlation was low (area under the curve [AUC] = 0.655). Endothelial cell morphology lacked any predictive power (AUC = 0.597). The clinical outcome regarding visual acuity seemed to be largely independent from both corneal endothelial cell density and morphology. Sub-analyses on transplanted patients stratified for their diagnoses vindicated these findings.

**Conclusions:**

Higher endothelial density (above a cut-off level of 2000 cells/mm^2^), as well as better endothelial morphology do not seem to be critical for transplant-corneal functionality in organ culture and up to 2 years after transplantation. Comparable long-term studies on graft survival are recommended to determine, whether the present endothelial density cut-off levels might be too stringent.

**Supplementary Information:**

The online version contains supplementary material available at 10.1007/s00417-023-06079-0.


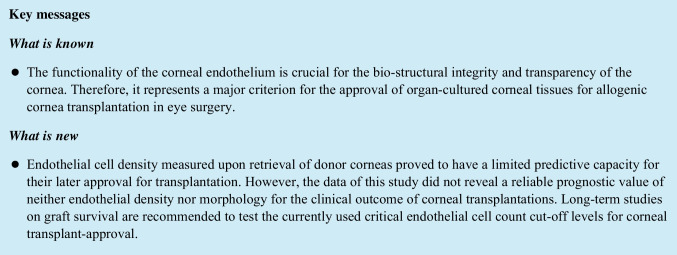


## Introduction

Corneal diseases are a major cause of visual impairment and blindness worldwide. According to the estimations of the World Health Organization (WHO), corneal opacities are the 3^rd^ most common cause of blindness worldwide, accounting for 7% of the world blind population [1-3].

Over a century after being first established as a surgical procedure, allogeneic cornea transplantation still plays an important role as a remedy of last resort for a great variety of corneal conditions. It is the most common type of solid tissue transplantation worldwide: about 150,000 keratoplasties are performed worldwide every year, predominantly in North America and Europe .

A high transplant-quality — mainly defined by biomechanical integrity and transparency — is essential for the long-term success of the treatment of corneal diseases by keratoplasty. One of the key factors is the functionality of the corneal endothelium, as it regulates nutrition and osmotic equilibrium of the cornea, thus warranting corneal transparency. The correlation between endothelium cell density (ECD) and functionality is well known and has been studied in detail [4-6].

Several factors have been studied for a possible influence on the ECD in donor corneas such as donor diseases, cause of death, corneal retrieval techniques and proficiency of the staff performing the microsurgery procedures [7, 8]. However, only donor age turned out to have a significant effect on the ECD of corneal transplants: statistically, ECD decreases throughout life by 0.5 to 0.6% per year [9, 10].

In functional corneal endothelium, mainly hexagonal, but also pentagonal cells are prevalent, as their geometry allows a mathematically economic, homogenous cover of the Descemet membrane following the corneal curvature [11]. In case of apoptotic or necrotic degradation of endothelial cells (due to increasing age or harmful environmental influences), neighboring endothelial cells increase their size to fill in any gaps in the endothelium layer, as they are unable to undergo mitosis in adult individuals [12, 13]. In consequence, these neighboring cells lose their hexagonal or pentagonal shape and become polygonal. Thus, the proportion of hexagonal and pentagonal cells in relation to polygonal cells decreases with the declining endothelium cell density resulting from cell degradation. While this correlation is well understood, it is obvious that morphology is not exclusively dependent on cell density: independent cellular- and molecular-biological effects emerging during normal (cell-) aging reducing the endothelial functionality also may affect endothelial morphology — and thus on the suitability of the corresponding corneas intended for transplantation.

New semi-automated methods of microscopic assessment of donor cornea endothelium not only allow the standardized quantification of mean cell density, but also facilitate an easier assessment of the morphology data of endothelial cells. Nonetheless, to our knowledge, no surveys have studied whether the endothelium cell morphology itself may have a cell-density-independent effect on the stability of human donor corneas in culture or on the clinical outcomes after transplantation.

The objective of this study is to explore whether endothelial morphology data of donor corneas (obtained after donation and during organ culture) could serve as an independent predictive parameter for the functional stability during in vitro cultivation and the clinical outcome after transplantation; this is done in comparison with data on the endothelial density in donor corneas, the actual main discriminative parameter for transplantation approval of donor corneas.

## Materials and methods

This retrospective case study was conducted at the Hamburg Eye Bank (HEB).

For the purpose of this study, we analyzed 1031 donor corneas that were processed in the Hamburg Eye Bank between 2012 and 2016. For this study, endothelial cell counts and endothelial cell morphology of the initial and the final examinations of donor corneas (see below), cultivation data (as the retrieval method, the time between donor death and cultivation of the cornea, the total duration of the cultivation, days in dextran-free and dextran-containing media) and donor data (donor age, donor death cause, basal diseases) were assessed for all corneas.

All corneal tissue was retrieved from deceased donors by the department of forensic medicine of the University Hospital Hamburg. Consent calls with relatives of the donor were performed in accord with the current law. Cornea recovery was performed by the medical forensic staff or by the trained technicians of the HEB. Tissue processing, preparation, and cultivation were performed by trained technicians of the HEB.

### Tissue retrieval

The retrieval of the donor cornea was performed by removing the corneoscleral disc in situ, without enucleating the eye. Both eyes of the donor were disinfected with PVP–iodine for 5 min and subsequently washed with 500 mL sterile, physiologic sodium chloride solution. After removing the conjunctiva using a sterile pair of Westcott scissors, the corneoscleral discs (CDs) were retrieved from the donor eyes using a 16-mm trephine. The excised CD was transferred to 12 mL sterile primary culture medium.

### Tissue cultivation

Organ cultivation was performed according to the methods established by Schroeter and Rieck [14]. Summarized description of the procedure follows: CDs were placed in sterile Boehnke-forks, submerged in culture flasks with supplemented cell culture medium (Minimum Essential Medium Eagle with Earle’s salts, 2% fetal bovine serum; 10 ml penicillin/streptomycin 10,000 U/10.000 µg/ml; 10 ml amphotericin B 250 µg/ml; 2 mM L-glutamine; 12.5 mM HEPES buffer; 0.22% sodium bicarbonate) and stored in an incubator (37 °C; 4% CO_2_) for up to 4 weeks. Media were exchanged once a week. All used media were checked for contamination. Only CDs with proven sterility of feeding media, functional endothelium (documented by final microscopy examinations), unsuspicious donor serology and medical history were approved for transplantation. At least 24 h before transplantation, CDs were placed in cell culture medium (s.a.) supplemented with 6% dextran 500 for de-swelling.

### Endothelium quality assessment

**Fig. 1:**
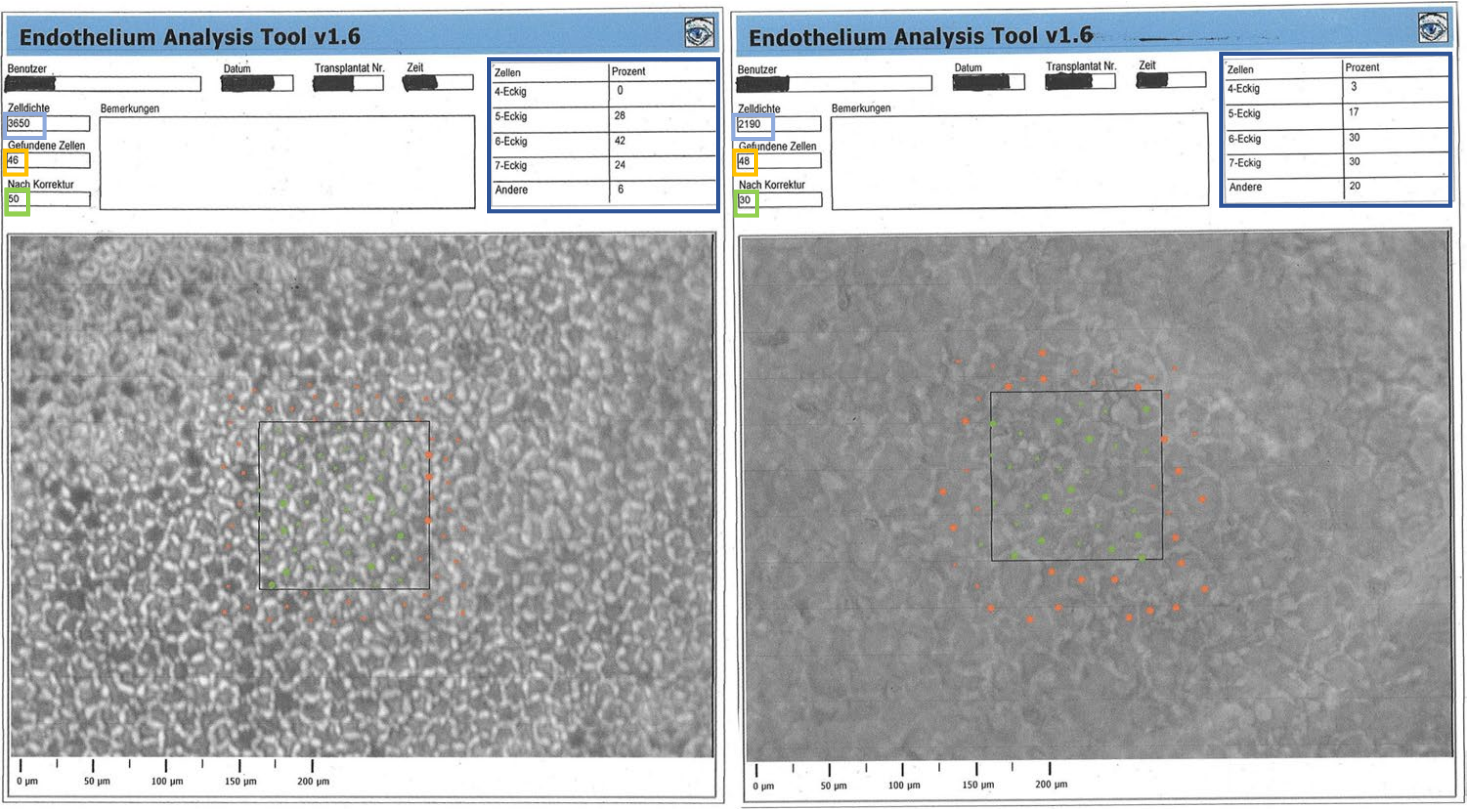
Anonymized printouts of exemplary semi-automated EAS-analyses on two corneas analyzed in our study. The left analysis shows a high quality endothelium, the right one an endothelium of poor quality. Automatically identified cells (marked by green or red dots within the target square of the microscopic photograph) before and after individual correction (numbers labeled by yellow and green frames respectively) and the cell density (based on corrected cell numbers [green dots] in cells/mm^2^; labelled in light blue frames) are shown at the upper left sides of the printouts, while morphology data (percentages of x-edged cells [green and red dots] after correction; labelled in dark blue frames) are shown at the upper right sides of the print outs

Analyses of the clarity of the corneas and endothelial quality assessments were performed by macroscopic and microscopy examinations of the CDs being immersed in balanced salt solution for the duration of the examination. Endothelial cells were counted for the first time immediately after retrieval of the CD in the eye bank directly before cultivation. Thereafter, at least two additional examinations of the corneal endothelium were performed: before de-swelling in dextran-containing medium (1 to 2 days before transplantation), and after de-swelling for at least 8 h (before transport into the operating theatre). To determine endothelium cell densities (ECDs), the cornea first underwent osmotic preparation in balanced salt solution for 60 s to achieve optimal visibility of the cells, after which the cornea was placed under a binocular inverted phase-contrast microscope (Nikon Eclipse Ti; 200-fold magnification) and photographed. The image was then transferred to the semi-automated EAS software (Robin Solutions, Hahn, Germany). The automatic analysis function was used (this quantification tool is regularly calibrated on-site). In this mode, the software determines the mean endothelial cell density (endothelial cells/mm^2^) and stratifies the measured cells according to their form in pentagonal, hexagonal and heteromorphic cells (percentage of hexa- and pentagonal cells; Fig. 1 illustrates an exemplary measurement of two corneas). Each automated cell density and morphology examination was checked (and if needed manually corrected) by an experienced examiner.

### Clinical development assessment of transplanted patients

For this study, objective refraction and visual acuity of transplanted patients approx. 3, 6, 12, 18 and 24 m (+ / − 1 month) after transplantation were analyzed. For each time-point group, median visual acuity and objective refraction values respectively were determined. Then, patients were grouped to a more- or less favourable clinical status depending on their individual value being above or below their common time-point mean value.

### Descriptive data of the analysed collectives

#### Cornea donor data

The mean donor age was 66.1 years (Range–Range, SD ± 14.4). There was data on the gender of 513 donors of whom 322 were male and 191 female, yielding a gender ratio of 1.68:1. Further data (death causes, average death-to-explantation intervals) are given in Table 1.

**Table 1:** Descriptive donor data

Cornea donors (between 2012 and 2016)	Data
N	516
Average age [years; SD]	66,1 (+ / − 14,4)
Donors with defined sex	513
Male	322
Female	191
Donors with defined death cause	404
Acute intoxication (alcohol, drugs, carbomonoxide)	12
Cardiopulmonal failure	254
Cerebral failure	35
Malignancy	9
Multi-organ failure	5
Politraumata	10
Sepsis	15
Suffocation / hanging	15
Other defined death cause	49
Average Death-to-explantation interval (DEI) [hours; SD]	41 (+ / − 17,7)

#### Clinical data after transplantation

Clinical data of 202 patients who underwent corneal transplantation from 27.06.2012 until 12.10.2016 using grafts that underwent the automatic endothelial counting and stratification was analyzed. Table 2 shows the descriptive data of graft recipients including the diagnoses and the condition being treated and the suturing method used.

**Table 2:** Descriptive data of graft recipients

Cornea recipients (between 2012 and 2016)	Data
N	202
Average age [years; min—max]	52 (11 to 92)
Recipients with defined sex	202
Male	105
Female	97
Diagnoses (of recipients) causing transplantation	202
Keratoconus	76
Keratitis	13
Ulcus	19
Fuchs Dystrophy	38
Corneal scars	25
Corneal distrophy	16
Bullous keratitis	15
Surgery data	202
Surgeons	5
Trephine size donor CS-disc [mm; min—max]	7.46 (6.25 to 8.50)
Trephine size recipient eye [mm; min—max]	7.20 (6.00 to 8.00)
pKPs (elective or á chaud)	178
DMEKs	24
Defined suture	194
* Single continued	46
* Double continued	108
* Single knots	40

#### Endothelial density and morphology data in organ culture

Overall, the endothelial density (ECD) and morphology (ECM) of 853 corneas could be determined at introduction in organ culture; from these, 556 corneas still displayed measurable ECDs and ECMs at final examination before their transplantation or discard. Table 3 gives descriptive information about their *mean initial (first measurement after retrieval) ****ECD**** and ****ECM**** values* and their *stabilities in culture* expressed by the relative losses in culture per day* (****rel.ECD-loss/d**** and ****rel.ECM-loss/d**** respectively)* and respective statistical parameters.

**Table 3:** Descriptive corneal endothelial density and morphology data (numeric)

Corneas (1031 corneascleral discs organ-cultured between 2012 and 2016)	N	Avg	SD	CI (95%)		Mean	Interquartile range	Distribution pattern
				Lower limit	Upper limit			
Endothelial cell density (ECD) [cells/mm^2^]	853	2640	421	2611	2669	2701	365	Non-parametric
ECD loss in culture (ECD-loss) [endothelial cell loss over complete cultivation time]	556	280	355	251	310	292	438	Non-parametric
ECD loss in culture per day (ECD-loss/d) [endothelial cell loss per day cultivation time]	556	17.5	28.6	15.2	19.9	15.4	24.2	Non-parametric
Realtive ECD loss in culture per day ( rel.ECD-loss/d) [% endotjelial cell loss per day cultivation time]	556	0.61	1.17	0.51	0.71	0.58	0.86	Non-parametric
Endothelial cell morphology (ECM) [% penta- and hexagonal cells]	853	55.6	14.0	54.7	56.5	57.0	13.0	Non-parametric
ECM loss in culture (ECM-loss) [% over complete cultivation time]	556	3.7	15.7	2.4	5.0	4.0	17.0	Non-parametric
Relative ECM loss in culture per day (rel.ECM-loss/d) [% morphology-loss per day cultivation time]	556	0.39	1.80	0.24	0.54	0.41	1.56	Non-parametric

### Statistics

Statistics and graphs were created by Microsoft-Excel and SPSS. Descriptive statistics comprised means, standard deviations, confidence intervals, medians and inter-quantiles. As the distribution patterns of the endothelial cell density and morphology variables turned out to be non-parametric (tested by Kolmogorov-Smirrnov-Tests and histograms; not shown), correlation analyses were done using Rho-Spearman tests while corresponding group comparisons were tested by nonparametric Mann–Whitney-*U* analyses. *P*-values > 0.05 were assigned as significant.

ROC-analyses were applied to compare predictive capacities (prospective sensitivity and specificity) of endothelial cell density and morphology on transplantation release (yes or no) or on more- or less favourable clinical status of transplanted patients (visual acuity and objective refraction) at specified time-points after transplantation. A central reference in ROC curves is the *area under the curve* (AUC), whereas a value of 0.5 indicates the complete failure of the tested predictor (hit probability equals to the flip of a coin), while a value of 1 indicates a perfect prediction.

## Results

### Correlations between donor age, ECD, ECM and their stabilities in organ culture (relative losses per day)

The descriptive data Tables 1, 2 and 3 displayed that our collectives (donors, recipients and transplant corneas) represent typical distributions of age, sex, death causes, clinical histories, etc. or cornea characteristics found in donor, medical or eye-banking data bases. To test the numeric variables donor age, death-to-explantation interval (DEI), initial endothelial cell density (ECD), initial endothelial cell morphology (ECM) and their respective stabilities in culture (relative ECD-/ECM-losses per day organ culture) for possible inter-correlations, a correlation matrix was done as shown in Table 4. Here, strong negative correlations could be demonstrated between donor age and ECD as much as ECM. At the same time, positive correlations were found between ECD, ECM and their respective stabilities in culture per day. In contrast, donor sex or death cause did not display any independent correlation with these variables (data not shown, see Supplementary Figs. 1–2).

**Table 4:**
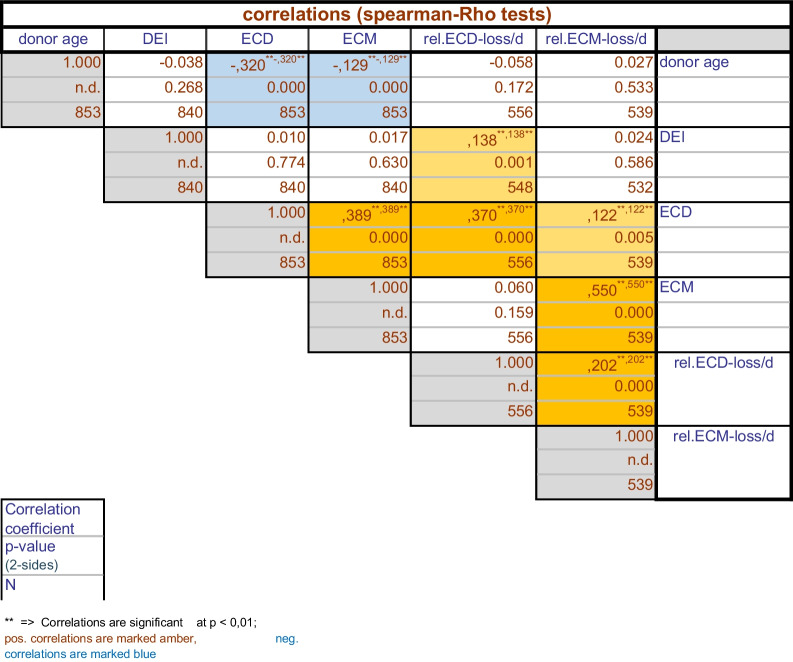
Correlation matrix of corneal endothelial density and -morphology data and donor age. Note that boxes with significant negative correlations are labelled in blue, while significant positive correlations are labelled in amber (light or deep for low or high correlation coefficients respectively)

It is logical and widely known amongst cornea researchers that endothelial morphology and density correlate with each other; this was vindicated by our own analysis (Table 4).

Thus, to prevent an interference of this correlation in our comparisons, we applied ROC analyses to compare the individual predictive capacities of initial ECD, initial ECM and their relative losses in organ culture per day on the selection of corneas for transplantation and their post-transplantation development in the following analyses.

### Predictive capacities of ECD and ECM and their stabilities in culture on selection of corneas for transplantation

**Fig. 2:**
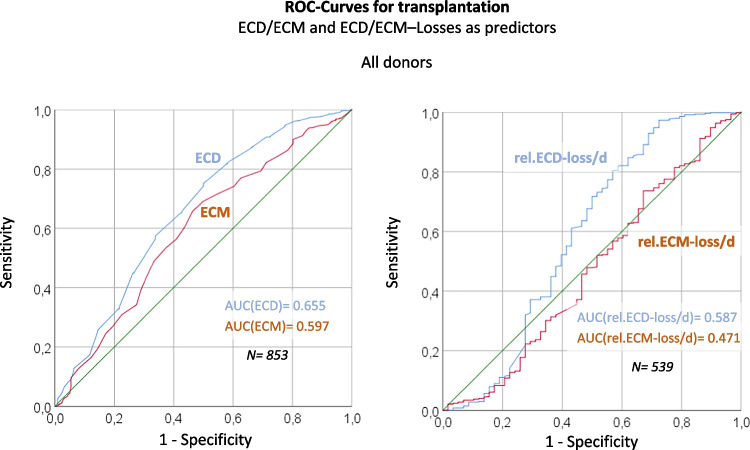
**Initial ECD/ECM and ECD/ECM–stabilities (= relative losses per day) in organ culture as predictors for transplantation release**. ROC-Curves. Note that the *areas under the curve* values (AUCs; a value of 0.5 indicates the complete failure of the tested predictor, a value of 1 indicates a perfect prediction) are indicated within the graphs

As shown in Fig. 2, the initial ECD displays a predictor of just moderate specificity and sensitivity (area under the curve [AOU] = 0.655) for the selection of the respective corneas for transplantation (or discard) at the end of organ culture. However, it still beats the corresponding predictive capacity of the initial ECM (AOU = 0.597). The ECD stability (relative loss of ECD in culture per day) is an even worse predictor (AUC = 0.587), while the ECM stability (relative loss of ECM in culture per day) bares any predictive power (AUC = 0.471). The same analyses applied on a subgroup comprising corneas from donors of 80 years or older only (which usually are prone to be discarded at the end of organ culture) showed that the predictive capacity of initial ECD and ECM for their selection for transplantation was somewhat higher (AUC values of 0.79 and 0.739 respectively; not shown, see Supplementary Fig. 3); note that (once again) ECD represents the better predictor then ECM.

### Predictive capacities of ECD and ECM and their stabilities in culture on the clinical developments of transplanted patients

**Fig. 3:**
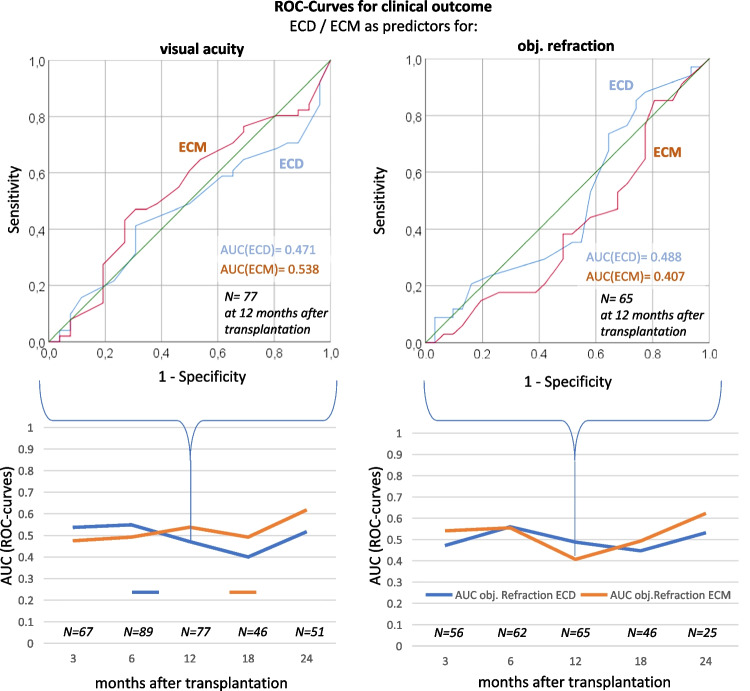
**Initial ECD/ECM as predictors for the clinical outcome.** ROC-curves. *Above:* Individual visual acuity (left graph) and objective refraction (measured light refraction by the cornea; right graph) 12 months after transplantation are displayed. The *areas under the curve* values (AUCs; a value of 0.5 indicates the complete failure of the tested predictor, a value of 1 indicates a perfect prediction) are indicated within the curves. *Below:* AUCs of the ROC-curves of all assessed tests are depicted as a function of time post transplantation (months)

The examination whether initial ECM and ECD of corneas would be a suitable predictive parameter for the clinical outcome after transplantation is depicted in Fig. 3: as shown, ECD and ECM measurements did not display any significant predictive value 12 months after keratoplasty on the visual acuity (AUC = 0.471 and 0.538 respectively) nor on the objective refraction (AUC = 0.488 and 0.407 respectively) of the transplanted individuals. This observation is representative for the complete observation period from 3 months till 24 months after transplantation (see lower part in Fig. 3). Correspondingly, the ECD-/ECM-stabilities (relative ECD- and ECM losses during culture per day) did not display any predictive power on the development of the visual acuity (AUC = 0.475 and 0.491) and objective refraction (AUC = 0.588 and 0.537) after their transplantation respectively) 12 months after transplantation and over the complete observation period (result not shown, see Supplementary Fig. 4).

To test whether diverging diagnoses (see Table 2) of the transplanted patients could mask or occult possible effects of endothelial density and morphology or their development in culture on the clinical outcome after transplantation in our main analyses, we made comparative ROC curves and non-parametrical Mann–Whitney-Tests on two subgroups of transplanted patients defined by each one defined diagnosis — keratoconus or Fuchs’ endothelial dystrophy. Still, for both diseases, no significant predictive effect of endothelial density or morphology or their stabilities in culture could be determined (details not shown, see table in the Supplementary Table).

## Discussion

One of the main problems in corneal transplant-surgery is the scarcity of suitable donor corneas [15]. Selecting donor corneas for transplantation is still mainly based on the absence of optical opacities and especially on sufficiently high endothelial cell densities (ECD), as a proper function of the endothelial cell layer is crucial for nutrition, osmoregulation, transparency and structural integrity of the cornea.

A regular hexagonal pattern provides the most stable covering of a plane and a deviation from this pattern may reflect a less stable monolayer: irregular shapes cause greater surface tension on the monolayer and cause a suboptimal geometric and thermodynamic configuration thus reducing the ability of corneal endothelium to maintain corneal homeostasis [16, 17].

The principal aim of this study was to examine whether endothelial cell morphology (ECM) could be used as a supplemental parameter to estimate the prospective quality of donor corneas after organ culture — and thus their suitability for transplantation.

A unique feature of our study is the additional analysis of clinical data obtained after the transplantation of the donor corneas. To our knowledge, this has not been done so far.

It is obvious that such analyses face several challenges:

First, the base collectives (donor characteristics, donated transplant-corneal ECD/ECM attributes and clinical data of transplanted patients) used for the comparisons should be widely homogeneous. Although there was a prevalence of male donors in our study group (1.68 to 1; Table 1), we found no difference either in ECD or in ECM between both sexes; also, death causes did not display any independent correlation with these variables (data not shown, Supplementary Figs. 1–2). These findings correspond well to previous studies [8; 19]. Donated transplant-corneal ECD/ECM attributes in organ culture (Table 3) also display homogeneous distribution patterns. However, the collective of cornea-transplanted patients is (inevitably) more heterogeneous (Table 2); this point may complicate the interpretation of our results (see below).

Second, we could demonstrate that a parameter of the analyzed collective — donor age — is associated with the compared variables ECD and ECM: The higher the donor age, the lower the corresponding ECD- and ECM values (see the respective correlation in Table 4). This is a well-known fact already demonstrated in previous studies [8; 20–23]. The average age of donors in this study was 66.1 years old, and only 5% of all corneas were retrieved from donors younger than 40 years of age. Thus, our conclusions on the results of our analysis might only apply to corneas from donors of elevated ages.

Third, we compared two parameters — ECD and ECM (and their stabilities in culture) — which can be assumed to influence each other; we indeed demonstrated that these parameters are correlated positively: the higher the corneal ECDs, the better the corresponding corneal ECMs (the same applies for their stabilities in culture; Table 4). Comparative analyses on the predictive capacities of these parameters have to consider their correlation.

Now, comparative ROC analyses represent a mathematically elegant method to compare the predictive powers of ECD and ECM (or their stabilities in culture) despite their evident correlation: as their individual areas under the curve (AUCs) always include the mutual correlation, differences of the AUCs necessarily are independent from it. A higher AUC of one of these variables (f.e. ECD or ECM) thus could indicate a higher predictive power which is independent from the other variable.

The main findings of our study are as follows:

Firstly, initial ECD or ECD development in culture displays only a limited predictive power (AUC of 0.655 or less; Fig. 2) for the approval (or rejection) of a donor cornea for transplantation.

Secondly, initial ECD or ECD development in culture does not have any predictive power (AUC values nearby 0.5; Fig. 3) for the clinical development after transplantation.

Thirdly, in no case — neither in organ culture before approval for transplantation nor in clinical development after transplantation — did initial ECM or ECM development in culture displays a predictive power superior to that of ECD or ECD development in culture (Figs. 2 and 3).

In summary, we could demonstrate that the initial ECM (measured after retrieval from the donor) as well as the ECM development during organ-culture do not provide any additional information to estimate the suitability of donor corneas for transplantation or their visual performances after transplantation — at least when semi-automated measurements were employed.

At the same time, the relatively low predictive power of initial ECD of donor corneas for transplantation-suitability is somewhat surprising. Even more, initial ECD lacks any prediction ability for the visual performance after transplantation. A possible explanation (at least for the latter observation) is that the usual cut-off level of corneal ECDs at their final assessment (at the end of organ culture) for transplantation approval (or against) in our institution lies at minimum 2000 cells/mm^2^ (for deep lamellar transplantation even at 2500 cells/mm^2^). Now, corneal endothelium usually still is functional at ECDs of 600 to 800 cells/mm^2^ [6]; thus, the elevated final ECD cut-off levels of 2000-/2500 cells/mm^2^ for transplant-cornea approvals could be too high to enable a detection of a putative predictive power of initial ECD for visual performance after transplantation in our study setting (i.e. within 2 years of observation). Noteworthy, in side analyses on sub-collectives from donors older than 80 years (with generally lower ECD distributions), the predictive capacity of initial ECD for transplantation approval proved to be somewhat higher (AUC = 0.796, not shown, Supplementary Fig. 3). In consequence, our observations of course do not categorically exclude an influence of initial endothelial density on the final approval for transplantation or the clinical outcomes of transplanted corneas. The poor predictive capacity of this variable in our analyses however highlights the need for further studies on the issue, whether current cut-off levels for transplantation approval could be downscaled; especially long-term observational studies on cornea-transplanted patients (longer than 2 years post transplantation) would be interesting.

In this study, high numbers of donors, transplant-corneas and (to a lesser extent) transplanted patients were analyzed thoroughly. However, its results and conclusions still need to be tested carefully in future surveys, as our study has some limitations.

The first obstacle is a consequence of the nature of semi-automated morphology analyses; it can only be as accurate, as the software performing it. In its current version, the EAS system used in this work mainly quantifies the number of 6- and 5-edged cells compared to pleomorphic cells. Some phenomena as cell agglomeration or degenerative aspects were not considered. A new version of the software will improve accuracy in the future.

The second limitation is that we measured the clinical outcome of corneal transplantation by comparing postoperative visual acuity and objective refraction. It is well known that there are a multitude of factors influencing these parameters — such as concurrent (known and unknown) ocular pathologies, subjective collaboration of the patient, size and position of the transplant, etc. While we presumed that — given a sufficient number of outcomes analyzed — these effects could cancel each other out, we admit that only a robust predictive corneal-quality marker could overcome all of these effects. This in mind — and under the premise that the main interfering factor might be the diagnostic background of the eye — we repeated the post-clinical comparative ROC analyses on two diagnostic groups (results not shown, see Supplementary Table). The results of these sub-analyses support the main observations and indicate that the poor/lacking predictive capacities of initial ECD/ECM (or their stability in organ culture) on the clinical outcomes of transplanted patients might be largely independent from their diagnosis. However, due to low (analyzable) case numbers, the reliability of these sub-analyses should be viewed critically — more comprehensive analyses are needed to test these observations.

Third, the vast majority of analyzed corneas was transplanted by penetrating keratoplasty (*n* = 178). In contrast, only 24 posterior-lamellar keratoplasties (DMEKs) were performed (see Table 2). Of course, this numerical imbalance does not allow reliable statements on putative differences of predictive powers of endothelial variables between these transplantation methods.

Finally, due to the retrospective setup of this study, we did not dispose on comprehensive data on corneal ECDs and ECMs after their transplantation. This data certainly is of considerable value and should be assessed in further studies.

## Conclusion

In conclusion, we state that the initial endothelial cell density of donor corneas assessed before organ culture cultivation remains the only — and just limited — predictor for their final approval for transplantation, while the endothelial cell morphology seems to have no quantifiable importance. In contrast, the clinical outcome of visual acuity up to 2 years after transplantation could be less dependent from endothelial cell density (and morphology) than expected. These observations could suggest considerations on less restrictive cut-off levels for the endothelial cell density for transplantation approval; however, further studies are needed.

## Supplementary Information

Below is the link to the electronic supplementary material.

Supplementary file1 (PPTX 108 KB)

Supplementary file2 (PPTX 94 KB)

Supplementary file3 (PPTX 116 KB)

Supplementary file4 (XLS 22 KB)

## Data Availability

Data not included in the manuscript will be made available on request.

## Code availability

The software application code is proprietary to the commercial supplier and not available to the authors.
