# Supplementary figures and images for: Semiautomatic assessment of endothelial density and morphology in organ-cultured corneas — potential predictors for transplantation suitability and clinical outcome?
Source: Graefes Arch Clin Exp Ophthalmol. 2023 Apr 28;261(9):2593–602. doi: 10.1007/s00417-023-06079-0 (PMC10432362; doi:10.1007/s00417-023-06079-0)

## Slide 1
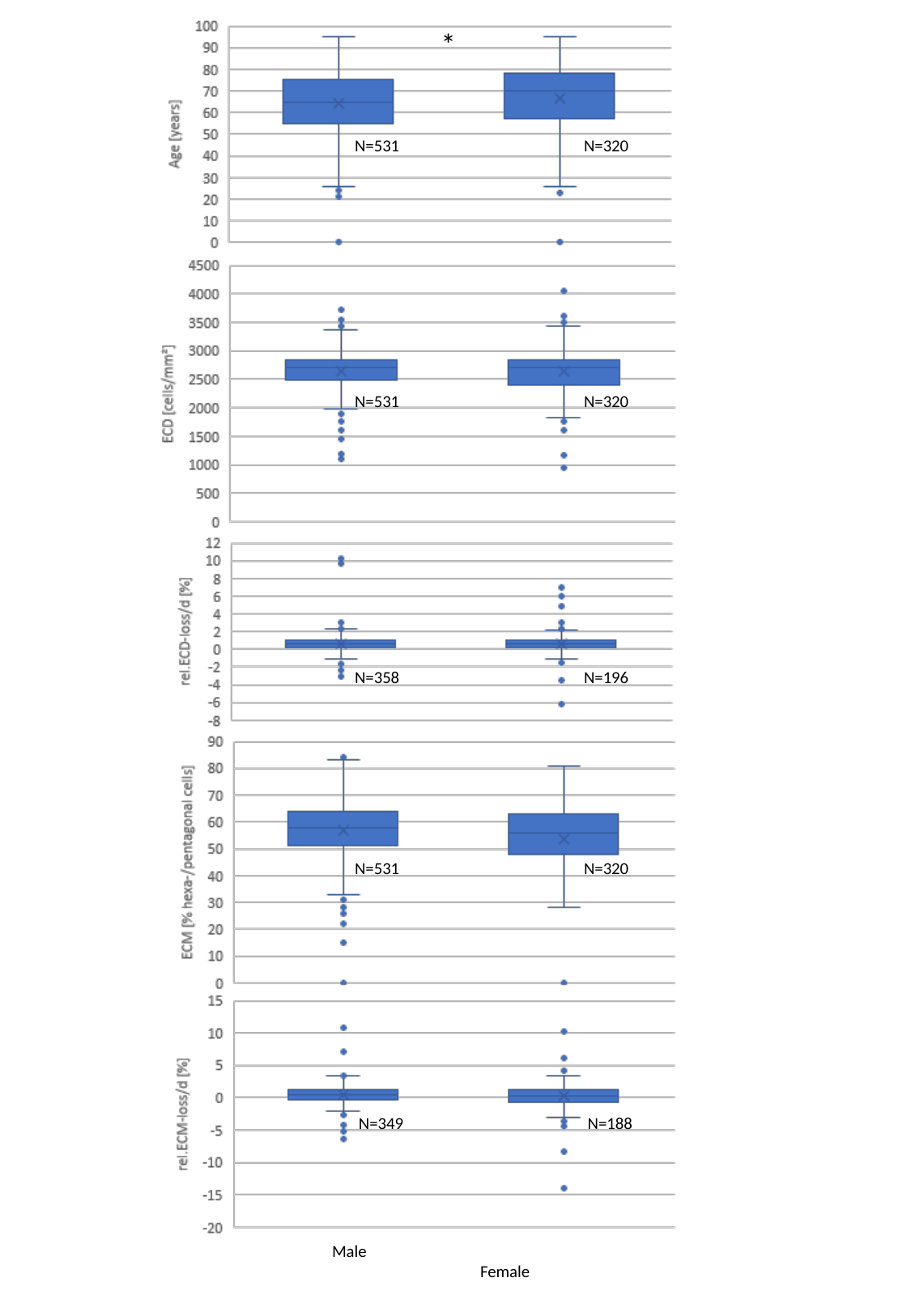

*
N=531
N=320
N=531
N=320
N=358
N=196
N=531
N=320
N=349
N=188
Male			 Female

Supplement: Supplementary file 1 — Supplementary file1 (PPTX 108 KB) [file 417_2023_6079_MOESM1_ESM.pptx]
