# Supplementary material for: Semiautomatic assessment of endothelial density and morphology in organ-cultured corneas — potential predictors for transplantation suitability and clinical outcome?
Source: Graefes Arch Clin Exp Ophthalmol. 2023 Apr 28;261(9):2593–602. doi: 10.1007/s00417-023-06079-0 (PMC10432362; doi:10.1007/s00417-023-06079-0)
Supplement: Supplementary file 2 — Supplementary file2 (PPTX 94 KB) [file 417_2023_6079_MOESM2_ESM.pptx]

## Slide 1
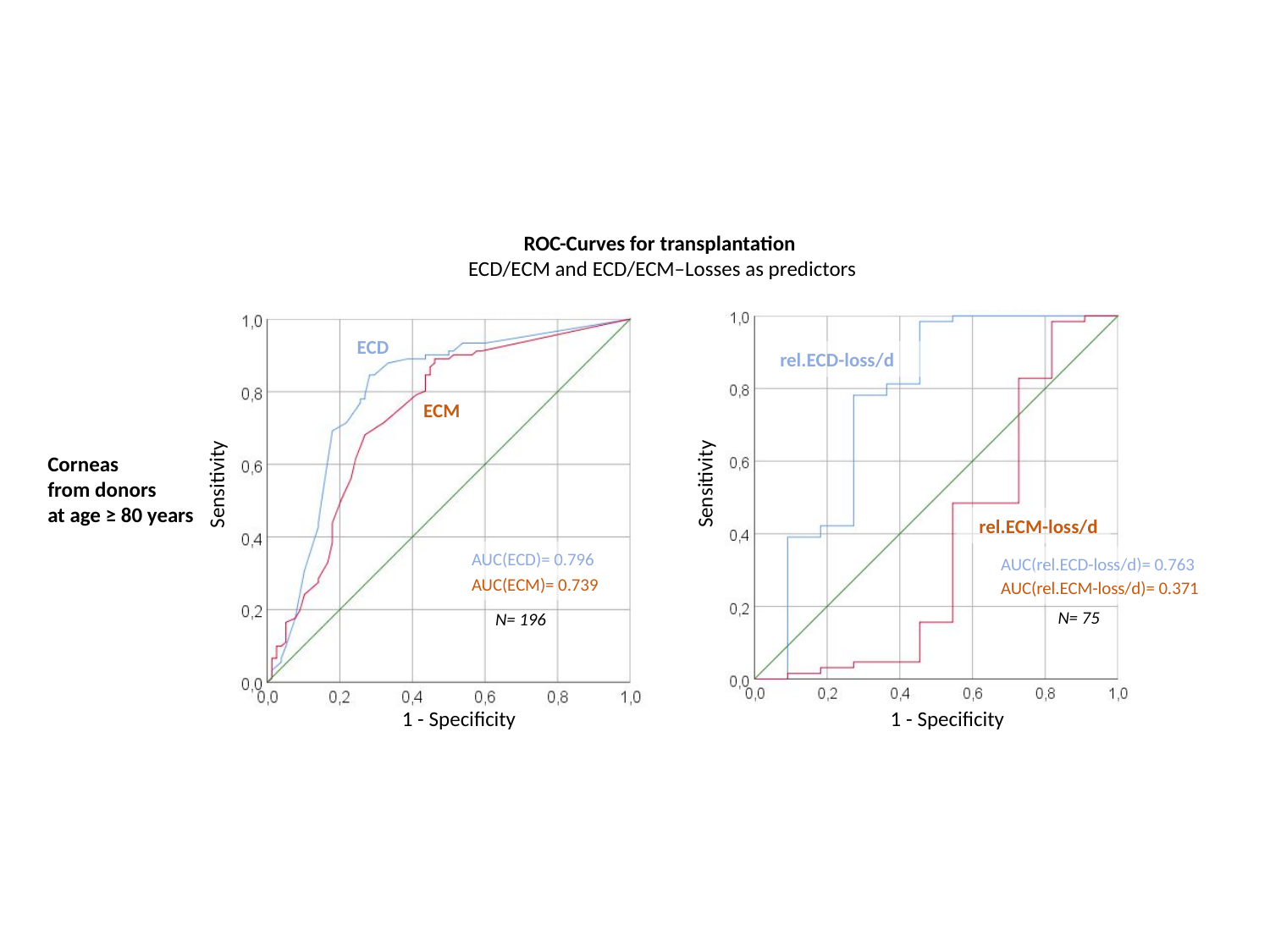

ROC-Curves for transplantation
ECD/ECM and ECD/ECM–Losses as predictors
ECD
rel.ECD-loss/d
ECM
Corneas
from donors
at age ≥ 80 years
Sensitivity
Sensitivity
rel.ECM-loss/d
AUC(ECD)= 0.796
AUC(rel.ECD-loss/d)= 0.763
AUC(ECM)= 0.739
AUC(rel.ECM-loss/d)= 0.371
N= 75
N= 196
1 - Specificity
1 - Specificity
