# Supplementary material for: Semiautomatic assessment of endothelial density and morphology in organ-cultured corneas — potential predictors for transplantation suitability and clinical outcome?
Source: Graefes Arch Clin Exp Ophthalmol. 2023 Apr 28;261(9):2593–602. doi: 10.1007/s00417-023-06079-0 (PMC10432362; doi:10.1007/s00417-023-06079-0)
Supplement: Supplementary file 3 — Supplementary file3 (PPTX 116 KB) [file 417_2023_6079_MOESM3_ESM.pptx]

## Slide 1
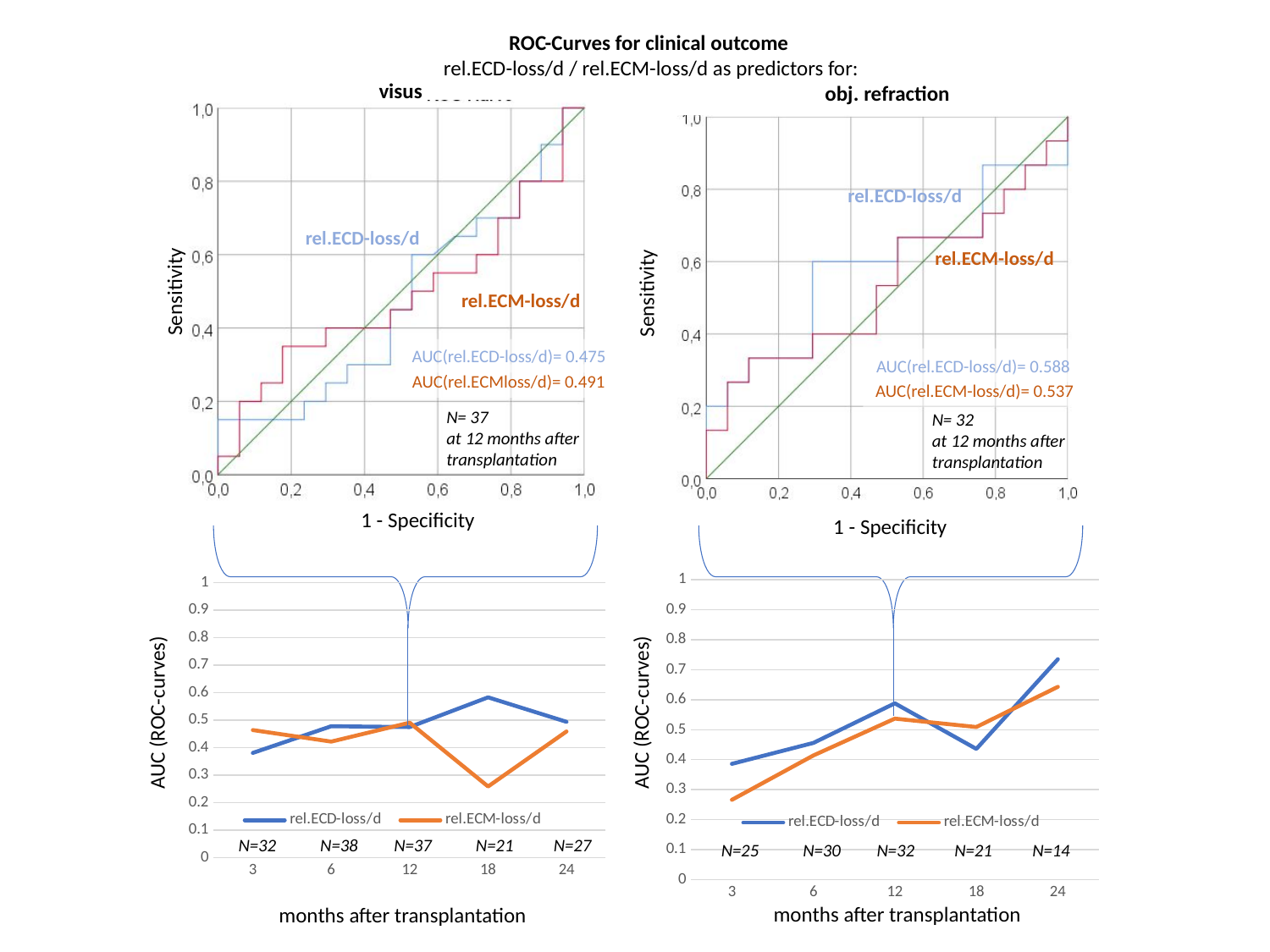

ROC-Curves for clinical outcome
rel.ECD-loss/d / rel.ECM-loss/d as predictors for:
visus
obj. refraction
rel.ECD-loss/d
rel.ECD-loss/d
rel.ECM-loss/d
Sensitivity
Sensitivity
rel.ECM-loss/d
AUC(rel.ECD-loss/d)= 0.475
AUC(rel.ECD-loss/d)= 0.588
AUC(rel.ECMloss/d)= 0.491
AUC(rel.ECM-loss/d)= 0.537
N= 37
at 12 months after transplantation
N= 32
at 12 months after transplantation
1 - Specificity
1 - Specificity
### Chart
| Category | rel.ECD-loss/d | rel.ECM-loss/d |
|---|---|---|
| 3 | 0.381 | 0.464 |
| 6 | 0.478 | 0.422 |
| 12 | 0.475 | 0.491 |
| 18 | 0.583 | 0.259 |
| 24 | 0.494 | 0.459 |
### Chart
| Category | rel.ECD-loss/d | rel.ECM-loss/d |
|---|---|---|
| 3 | 0.386 | 0.266 |
| 6 | 0.456 | 0.414 |
| 12 | 0.588 | 0.537 |
| 18 | 0.436 | 0.509 |
| 24 | 0.735 | 0.643 |AUC (ROC-curves)
AUC (ROC-curves)
 N=32 N=38 N=37 N=21 N=27
 N=25 N=30 N=32 N=21 N=14
months after transplantation
months after transplantation
